# Prevalence and determinants of depressive symptoms among community-dwelling older adults in China based on differences in living arrangements: a cross-sectional study

**DOI:** 10.1186/s12877-023-04339-6

**Published:** 2023-10-10

**Authors:** Chang Fu, Lianmeng Cao, Fan Yang

**Affiliations:** 1 Department of Health Service and Management, School of Public Health and Management, Binzhou Medical University, No.346 Guanhai Road, Yantai, Shandong 264003 China; 2 Department of Gastrointestinal Surgery Bariatric and Metabolic Surgery, Binzhou Medical University Hospital, No.661 2nd Huanghe Road, Binzhou, Shandong 256603 China; 3 Department of Information Center, Xiangyang No.1 People’s Hospital, Hubei University of Medicine, 15th Jiefang Road, Xiangyang, Hubei 441000 China

**Keywords:** Depressive symptoms, Older adults, Determinants, Living arrangement

## Abstract

**Background:**

Older adults with different living arrangements may have different mental health statuses and different factors that influence their mental health. The aim of the present study is to investigate the prevalence and determinants of depressive symptoms among community-dwelling older adults in China based on differences in their living arrangements.

**Methods:**

Participants were 6,055 older adults from the 2015 China Health and Retirement Longitudinal Study. Depressive symptoms and their determinants were evaluated using the 10-item Center for Epidemiologic Studies Depression Scale and multivariate logistic regression analysis, respectively.

**Results:**

The prevalence of depressive symptoms among older adults living alone, as a couple, and with children was 47.8%, 33.2%, and 39.5%, respectively. The common risk factors for depressive symptoms were shorter sleep duration, poorer activities of daily living, and poorer self-rated health. Women, those with lower educational levels, and those suffering from chronic diseases had a higher risk of depressive symptoms among older adults living as a couple and those living with children. Smoking and participation in economic activities were also risk factors of depressive symptoms among older adults living with children and those living alone, respectively.

**Conclusions:**

The findings suggest that older adults living as couples had the lowest prevalence of depressive symptoms, while those living alone had the highest prevalence of depressive symptoms. The determinants of depressive symptoms differed by living arrangement; hence, they should be considered in future interventions.

**Supplementary Information:**

The online version contains supplementary material available at 10.1186/s12877-023-04339-6.

## Background

Depressive symptoms are one of the most common mental health problems among older adults [1]. Furthermore, it can significantly increase older adults’ risk of frailty [2] and decrease their quality of life [3]. China has the largest population of older adults in the world; specifically, by the end of 2021, its population aged 60 years and above reached 267.36 million, accounting for 18.9% of its total population [4]. A systematic review and meta-analysis showed that the pooled overall prevalence of depressive symptoms among older adults in China was 20.0% [5]. The high prevalence of depressive symptoms among older adults has significantly increased the degree of medical expenditure in the country [6]. A study from China reported that there were about $42.67 per person of annual medical spending which is induced by depressive symptoms and depression [7]. Therefore, exploring the determinants of depressive symptoms and reducing their prevalence are crucial for older adults’ mental health. Previous studies reported several determinants of depressive symptoms, such as chronic diseases [8], educational level [9], and sleep duration [10].

In Chinese culture, living with aging parents and taking care of them is a primary moral principle for the younger generations [11]. However, with social economic development, including reduced fertility, the migration of the younger labor force from less developed to more developed areas, and the younger generation’s preference to live independently after marriage [12]—the living arrangements of older adults have changed rapidly in China. Recent years have seen the number of empty-nest older adults, which refers to those living alone or only with a spouse/partner owing to their children having already left home, increases rapidly [12]. By 2030, empty-nest older adults will account for 90% of the total older adult population in the country [13]. Therefore, the living arrangements of most older adults have shifted to either living alone, as a couple, or with children.

Previous studies have found that living arrangements are associated with older adults’ mental health outcomes, such as loneliness [14], suicidal ideation [15], and depressive symptoms [16] and that a lack of family emotional support or financial support from children may be vital for the poor mental health of empty-nest older adults [17, 18]. Accordingly, the mental health status of this population group has been concerned by many researchers. Studies found that empty-nest older adults are more likely to have depressive symptoms than non-empty-nest ones [19–21]. Some other studies have explored the determinants of depressive symptoms among empty-nest older adults, such as sex, education level, and chronic diseases [22, 23]. However, these studies did not divide the empty-nest older adults into those living alone or as part of a couple.

A previous study found that older adults living as a couple had better cognitive function than those living with children; the possible reason for this phenomenon could be related to these older adults having less burden related to caring for children or grandchildren and being able to enjoy their life as a couple [24]. Therefore, older adults living as a couple may have a better mental health status than those living alone or with children, and the determinants of mental health may differ by their living arrangement. By further classifying empty-nest older adults into those living alone and those living as a couple and exploring the determinants of depressive symptoms among the three groups, i.e., those living alone, as a couple, or with children, those being most in need of mental health improvement can be identified and a scientific basis for targeted community interventions and relevant health policies can be provided.

In this study, we investigate both the prevalence and determinants of depressive symptoms among older adults according to three different kinds of living arrangements. To obtain knowledge on this topic may allow invested stakeholders to ensure more precise decision-making regarding the implementation of interventions for depressive symptoms in the older adult population.

## Methods

### Study population

This study used data released by the National School of Development of Peking University from the 2015 China Health and Retirement Longitudinal Study, which comprised a nationally representative sample of middle-aged and older Chinese community-dwelling residents. This sample included 21,096 individuals, selected through multistage sampling from 12,400 families in 450 villages and 150 county-level units [25]. The inclusion criteria were individuals aged ≥ 60 years and who were able to communicate with the interviewers. The exclusion criteria were individuals with serious diseases, e.g., Alzheimer’s and serious psychiatric disorders other than depression and those who did not provide information on their living arrangements and/or were living in nursing homes [26]. Older adults living with others (except children or spouse/partners) were excluded, as they comprised less than 2% of the sample. Finally, data from 6,055 participants were analyzed.

The original CHARLS was approved by the Ethical Review Committee of Peking University. All participants were asked to write an informed consent for inclusion before participating in this survey. Secondary analysis of CHARLS data does not require ethics approval. All analyses in this study were performed in accordance with relevant guidelines and regulations, including the Helsinki Declaration revised in 1989.

### Measurement of depressive symptoms

Depressive symptoms were measured using the 10-item Center for Epidemiologic Studies Depression Scale (CES-D 10), which has good reliability and validity among older Chinese respondents [27]. Its 10 items were used to ask the respondents to rate how often they experienced depressive symptoms in the past week [25]. Four choices were selected to answer each question: zero (*rarely)*, 1 (*1–2 days)*, 2 *(3–4 days)*, and 3 *(5–7 days)*. The answers from respondents were recorded as 0 to 3 and 3 to 0 for the negatively and positively scored questions, respectively [28]. The total scores ranged from 0 to 30 [29], and a cut-off point of 10 was used for the diagnosis of depressive symptoms [27]. Cronbach’s alpha values for the CES-D 10 in this study were as follows: older adults living alone group, α = 0.680; living as a couple group, α = 0.637; and living with children group, α = 0.649.

### Assessment of living arrangements

The participants were asked the question: “Who do you live with now, or with whom have you lived for more than 11 months during the past year?” According to their answers, they were categorized as older adults living alone, as a couple, or with children [24].

### Measurement of determinants

In this study, the determinants of older adults’ depressive symptoms were chosen based on previous studies [30–34]. Participants’ demographic characteristics included age, sex, marital status, education level, and region of living (urban or rural). Their lifestyle factors included smoking (current/past or never), alcohol consumption (yes or no), and sleep duration (< 6 h, 6–8 h, and ≥ 9 h per day) [35].

The health status characteristics included chronic disease (had or did not have), activities of daily living (ADL), body mass index (BMI), and self-rated health. The abilities of eating, dressing, using the toilet, getting in and out of bed, defecating, and bathing were included to evaluate the participant’s ADL [36]. Cronbach’s alpha values for the ADL in this study were as follows: older adults living alone group, α = 0.798; living as a couple group, α = 0.764; and living with children group, α = 0.834. Participants who expressed difficulty with any of these activities were classified as having ADL functional decline. BMI was categorized as normal (18.5 ≤ BMI < 24 kg/m^2^), underweight (BMI < 18.5 kg/m^2^), overweight (24 ≤ BMI < 28 kg/m^2^), and obese (BMI ≥ 28 kg/m^2^) [37]. Self-rated health was divided into good, fair, and poor [38]. Participants were also asked whether they had participated in economic activities.

### Statistical analysis

Data were analyzed using SPSS version 20.0 (IBM Corp. Armonk, NY, USA). The multiple imputation was carried out using chained equations with 10 iterations to address missing data [39]. Based on the previous study, multiple imputation is the most accurate technique for dealing with missing values when assessing depressive symptoms [40]. In this study, the proportions of missing values were region of living (0.4%), education level (6.4%), smoking (0.5%), alcohol consumption (0.5%), sleep duration (8.1%), chronic disease (10.8%), ADL (22.1%), BMI (17.8%), self-rated health (5.1%), economic activity (0.7%), depressive symptoms (12.7%). There were no missing values for living arrangements, age, sex, and marital status. The logistic model was used to identify the variables predicting missing responses, which showed that the missing data of variables were related to the respondents’ age and the result indicated that the data were missing at random [40]. Therefore, the multiple imputation was carried out using chained equations with 10 iterations to address missing data [39]. Participants’ characteristics were described using percentages for categorical data. The prevalence of depressive symptoms was compared between the three groups by their characteristics using chi-square tests. Multivariate logistic regression analysis was used to examine the association between living arrangement and depressive symptoms and examine the determinants of depressive symptoms. Before data analysis, residual analysis was performed with logistic regression to test the assumptions of the regression models [41]. The variance inflation factor (VIF) was used to assess the multicollinearity of the variables, which showed that there is no muticollinearity in this study (VIF < 10) [42] (STable 1). The logistic regression model was evaluated by Hosmer-Lemeshow goodness-of-fit test and *p* > 0.05 was considered a well-fitting logistic regression model [43] (STable 2). Statistical significance was set at *p* < 0.05.

## Results

### Sample characteristics

Table 1 shows participants’ sociodemographic characteristics, health status, and depressive symptoms. Of the 6,055 participants, 58.4% were 60–69 years old, 54.3% were men, 66.4% were married/cohabitating, 79.7% lived in rural areas, and 54.3% had no formal education. Furthermore, 52.7% had never smoked, 65.7% did not consume alcohol, 45.1% slept 6–8 h per day, 82.7% had at least one chronic disease, 64.0% had normal ADL function, 52.1% had normal BMI, 51.2% had fair self-rated health status, and 50.6% participated in economic activities. The prevalence of depressive symptoms among the total sample was 38.6%, whereas its prevalence among older adults living alone, as a couple, and with children was 47.8%, 33.2%, and 39.5%, respectively. There were statistically significant differences in age, sex, marital status, region of living, education level, alcohol consumption, sleep duration, BMI, participation in economic activities, and depressive symptoms between the three groups (*p* < 0.05). After controlling all confounders, the results revealed that, compared with older adults living alone, older adults living as a couple and living with children were both less likely to have depressive symptoms (STable 3).

**Table 1:** Characteristics of the study population

Characteristics	Total(n = 6055)	Living alone (n = 1191)	Living as a couple (n = 2288)	Living with children (n = 2576)	*χ* ^*2*^	*P*
Age (%)					186.737	**< 0.001**
60 ~ 69	58.4	44.4	63.3	60.6		
70 ~ 79	30.3	37.5	30.4	26.7		
≥ 80	11.3	18.1	6.3	11.3		
Sex (%)					200.850	**< 0.001**
Male	54.3	42.1	65.3	50.2		
female	45.7	57.9	34.7	49.8		
Marital status (%)					2504.712	**< 0.001**
Married/ Cohabitating	66.4	16.0	99.3	60.4		
Never married/divorced/ widowed/ separated	33.6	84.0	0.7	39.6		
Region of living (%)					13.749	**0.001**
Urban	20.3	16.5	21.2	21.4		
Rural	79.7	83.5	78.8	78.6		
Education level(%)					151.580	**< 0.001**
No formal education	54.3	67.0	45.5	56.4		
Elementary school	23.3	18.9	27.0	22.0		
Middle school	14.0	9.2	16.5	14.0		
High school or above	8.4	4.9	11.0	7.7		
Smoking (%)					49.009	0.456
Current/past	47.3	42.2	53.0	44.7		
Never	52.7	57.8	47.0	55.3		
Alcohol consumption(%)					47.212	**< 0.001**
Yes	34.3	28.4	39.3	32.6		
No	65.7	71.6	60.7	67.4		
Sleep duration (hours per day), (%)					40.533	**< 0.001**
< 6	27.8	35.1	25.2	26.9		
6 ~ 8	45.1	38.5	47.6	45.8		
≥ 9	27.1	26.4	27.2	27.3		
Chronic disease(%)					0.382	0.826
No	17.3	17.3	17.0	17.7		
Yes	82.7	82.7	83.0	82.3		
ADL(%)					3.555	0.169
Completely normal	64.0	62.2	65.6	63.5		
Functional decline	36.0	37.8	34.4	36.5		
BMI(%)					25.492	**< 0.001**
Normal	52.1	56.1	50.1	52.1		
Underweight	8.2	8.1	6.9	9.3		
Overweight	29.2	25.9	30.9	29.3		
Obese	10.5	9.9	12.2	9.3		
Self-rated health (%)					6.680	0.154
Good	20.7	20.6	19.6	21.8		
Fair	51.2	49.1	52.6	50.8		
Poor	28.2	30.3	27.8	27.5		
Economic activity (%)					54.167	**< 0.001**
Yes	49.4	42.9	55.1	47.3		
No	50.6	57.1	44.9	52.7		
Depressive symptoms (%)					62.574	**< 0.001**
Yes	38.6	47.8	33.2	39.5		
No	61.4	52.2	66.8	60.5		

Note: BMI, body mass index; ADL, activities of daily living; Percentages may not add up to 100% due to rounding

### Prevalence of depressive symptoms based on different sociodemographic characteristics

As shown in Table 2, there were significant differences among older adults living alone in terms of sex, region of living, education level, smoking status, sleep duration, chronic diseases, ADL, self-rated health, and prevalence of depressive symptoms. There were also differences among those living as a couple in terms of sex, region of living, education level, smoking status, alcohol consumption status, sleep duration, chronic diseases, ADL, self-rated health, and prevalence of depressive symptoms. Furthermore, among those living with children, significant differences were noted in terms of sex, marital status, region of living, educational level, smoking status, alcohol consumption status, sleep duration, chronic diseases, ADL, BMI, self-rated health, and prevalence of depressive symptoms.

**Table 2:** Prevalence of depressive symptoms in different social-demographic characteristics of the study population

Characteristics	Living alone		*χ* ^*2*^	*P*	Living as a couple		*χ* ^*2*^	*P*	Living with children		*χ* ^*2*^	*P*
	Incidence	Non-case			Incidence	Non-case			Incidence	Non-case		
Age			3.735	0.155			1.695	0.429			2.035	0.362
60 ~ 69	50.9	45.3			65.6	64.1			64.9	67.0		
70 ~ 79	37.5	40.3			30.0	30.1			28.4	25.6		
≥ 80	11.6	14.4			4.4	5.8			6.8	7.3		
Sex			18.291	**< 0.001**			73.880	**< 0.001**			53.615	**< 0.001**
Male	36.6	50.0			52.4	71.4			43.3	59.3		
female	63.4	50.0			47.6	28.6			56.7	40.7		
Marital status			1.766	0.184			2.050	0.152			23.429	**< 0.001**
Married/ Cohabitating	15.9	19.1			98.9	99.4			58.8	69.0		
Never married/divorced/ widowed	84.1	80.9			1.1	0.6			41.2	31.0		
Region of living			16.718	**< 0.001**			34.889	**< 0.001**			24.117	**0.001**
Urban	11.7	21.3			14.0	25.3			16.7	25.7		
Rural	88.3	78.7			86.0	74.7			83.3	74.3		
Education level			24.675	**< 0.001**			76.182	**< 0.001**				**< 0.001**
No formal education	71.6	57.1			55.3	38.7			64.7	44.5		
Elementary school	18.2	22.8			26.8	27.3			19.8	25.5		
Middle school	6.7	13.0			13.0	18.9			10.8	19.3		
High school or above	3.6	7.0			4.9	15.1			4.7	10.7		
Smoking			12.226	**< 0.001**			23.141	**< 0.001**			11.008	**0.001**
Current/past	38.4	49.3			45.4	56.5			42.0	49.3		
Never	50.7	50.7			54.6	43.5			58.0	50.7		
Alcohol consumption			2.288	0.130			39.609	**< 0.001**			7.175	**0.007**
Yes	26.6	30.9			30.7	45.0			30.6	36.2		
No	73.4	69.1			69.3	55.0			69.4	63.8		
Sleep duration (hours per day)			38.280	**< 0.001**			100.624	**< 0.001**			82.444	**< 0.001**
< 6	45.2	26.4			38.6	18.3			37.0	19.9		
6 ~ 8	32.3	44.8			40.3	51.6			43.2	49.0		
≥ 9	22.5	28.9			21.1	30.1			19.9	31.1		
Chronic disease			17.014	**< 0.001**			38.940	**< 0.001**			40.858	**< 0.001**
No	12.4	22.9			20.6	9.3			10.6	21.8		
Yes	87.6	77.1			79.4	90.7			89.4	78.2		
ADL			57.477	**< 0.001**			105.089	**< 0.001**			110.954	**< 0.001**
Completely normal	50.6	76.1			51.2	76.1			53.1	77.6		
Functional decline	49.4	23.9			48.8	23.9			46.9	22.4		
BMI			2.089	0.554			6.691	0.082			10.062	**0.018**
Normal	55.0	57.2			51.7	48.5			53.1	50.7		
Underweight	6.9	8.5			8.2	5.9			10.3	7.1		
Overweight	27.5	25.7			29.2	32.7			27.1	32.4		
Obese	10.6	8.5			10.9	12.9			9.5	9.8		
Self-rated health			110.651	**< 0.001**			272.589	**< 0.001**			285.551	**0.033**
Good	11.4	28.1			7.1	25.5			10.4	29.5		
Fair	42.3	54.6			43.9	57.6			43.9	55.8		
Poor	46.3	17.3			49.0	16.9			45.7	43.9		
Economic activity			1.994	0.158			0.227	0.634			0.022	0.883
Yes	48.8	44.3			43.8	55.1			50.1	50.4		
No	51.2	55.7			56.2	44.9			49.9	49.6		

Note: BMI, body mass index; ADL, activities of daily living; Percentages may not add up to 100% due to rounding

### Determinants of depressive symptoms among older adults with different living arrangements

Among the older adults living alone, the results of multivariate logistic regression analysis showed that living in rural areas (odds ratio [OR] = 1.571, 95% confidence interval [CI]: 1.035–2.386), poorer ADL (OR = 2.375, 95% CI: 1.715–3.288), poorer self-rated health (fair: OR = 1.637, 95% CI: 1.127–2.380; poor: OR = 4.487, 95% CI: 2.895–6.955), and participation in economic activities (OR = 1.386, 95% CI: 1.006–1.911) were associated with a higher risk of depressive symptoms. Conversely, middle school education levels (OR = 0.580, 95% CI: 0.345–0.973) and longer sleep duration (6–8 h: OR = 0.437, 95% CI: 0.304–0.628; ≥ 9 h: OR = 0.542, 95% CI: 0.374–0.784) were associated with a lower risk of depressive symptoms (Table 3).

Among older adults living as a couple, the results showed that women (OR = 1.811, 95% CI: 1.340–2.449), having chronic diseases (OR = 1.439, 95% CI: 1.039–1.994), poorer ADL (OR = 2.127, 95% CI: 1.672–2.706), and poorer self-rated health (fair: OR = 2.282, 95% CI: 1.617–3.221; poor: OR = 6.690, 95% CI: 5.575–9.782) were associated with a higher risk of depressive symptoms. By contrast, higher education levels (middle school: OR = 0.676, 95% CI: 0.489–0.935; high school and above: OR = 0.452, 95% CI: 0.293–0.697) and longer sleep duration (6–8 h: OR = 0.484, 95% CI: 0.378–0.619; ≥ 9 h: OR = 0.445, 95% CI: 0.329–0.602) were associated with a lower risk of depressive symptoms (Table 3).

Among older adults living with children, the results showed that women (OR = 2.191, 95% CI: 1.640–2.927), having chronic diseases (OR = 1.399, 95% CI: 1.047–1.869), poorer ADL (OR = 2.290, 95% CI: 1.793–2.926), and poorer self-rated health (fair: OR = 1.937, 95% CI: 1.455–2.597; poor: OR = 5.806, 95% CI: 4.184–8.059) were associated with a higher risk of depressive symptoms. Conversely, being ≥ 80 years old (OR = 0.565, 95% CI: 0.339–0.942), higher education levels (elementary school: OR = 0.678, 95% CI: 0.529–0.869; middle school: OR = 0.572, 95% CI: 0.414–0.791; high school and above: OR = 0.440, 95% CI: 0.283–0.685), never having smoked (OR = 0.710, 95% CI: 0.535–0.944), and longer sleep duration (6–8 h: OR = 0.700, 95% CI: 0.534–0.919; ≥ 9 h: OR = 0.475, 95% CI: 0.360–0.628) were associated with a lower risk of depressive symptoms (Table 3).

**Table 3:** Multivariate logistic regression analysis on the influencing factors associated with depressive symptom among older adults with different living arrangements

Characteristics	Living alone		Living as a couple		Living with children	
	OR	*95%* CI	OR	*95%* CI	OR	*95%* CI
Age (ref. 60 ~ 69)						
70 ~ 79	0.734	(0.525,1.028)	0.862	(0.681,1.091)	0.933	(0.736,1.183)
≥ 80	0.634	(0.388,1.036)	0.672	(0.407,1.110)	0.565*	(0.339,0.942)
Sex (ref. male)						
Female	1.364	(0.897,2.073)	1.811***	(1.340,2.449)	2.191***	(1.640,2.927)
Marital status(ref. Married/Cohabitating)						
Never married/ divorced/widowed	1.119	(0.748,1.675)	1.721	(0.564,5.256)	1.148	(0.914,1.442)
Region of living (ref. Urban)						
Rural	1.571*	(1.035,2.386)	1.328	(0.962,1.833)	1.079	(0.795,1.464)
Education level(ref. No formal education)						
Elementary school	0.736	(0.497,1.090)	0.847	(0.645,1.113)	0.678**	(0.529,0.869)
Middle school	0.580*	(0.345,0.973)	0.676*	(0.489,0.935)	0.572**	(0.414,0.791)
High school or above	0.650	(0.319,1.323)	0.452***	(0.293,0.697)	0.440***	(0.283,0.685)
Smoking (ref. Current/past)						
Never	1.282	(0.873,1.882)	1.057	(0.807,1.386)	0.710*	(0.535,0.944)
Alcohol consumption(ref. Yes)						
No	0.911	(0.644,1.289)	1.156	(0.908,1.472)	0.856	(0.676,1.085)
Sleep duration(ref. < 6)						
6 ~ 8	0.437***	(0.304,0.628)	0.484***	(0.378,0.619)	0.700*	(0.534,0.919)
≥ 9	0.542**	(0.374,0.784)	0.445***	(0.329,0.602)	0.475***	(0.360,0.628)
Chronic disease(ref.No)						
Yes	1.411	(0.918,2.170)	1.439*	(1.039,1.994)	1.399*	(1.047,1.869)
ADL(ref. Completely normal)						
Functional decline	2.375***	(1.715,3.288)	2.127***	(1.672,2.706)	2.290***	(1.793,2.926)
BMI(ref. Normal)						
Underweight	0.796	(0.414,1.531)	0.966	(0.612,1.526)	1.234	(0.838,1.818)
Overweight	0.973	(0.641,1.477)	0.795	(0.610,1.036)	0.843	(0.641,1.110)
Obese	0.892	(0.487,1.637)	0.710	(0.503,1.004)	0.735	(0.521,1.088)
Self-rated health(ref. Good)						
Fair	1.637**	(1.127,2.380)	2.282***	(1.617,3.221)	1.937***	(1.455,2.597)
Poor	4.487***	(2.895,6.955)	6.690***	(5.575,9.782)	5.806***	(4.184,8.059)
Economic activity (ref. No)						
Yes	1.386*	(1.006,1.911)	1.157	(0.912,1.466)	1.141	(0.896,1.454)

Note: * p<0.05; **p<0.01; ***p<0.001; BMI, body mass index; ADL, activities of daily living

## Discussion

In this study, the prevalence of depressive symptoms and its determinants among older adults with different living arrangements were firstly compared. Our findings provide suggestions for interventions to improve the mental health of community-dwelling older adults.

Among the three evaluated groups, older adults living alone had the highest prevalence of depressive symptoms. One explanation is that older adults who live alone are more likely to suffer from loneliness [44], and higher loneliness levels are associated with higher depressive symptoms [45]. Another explanation is that the group of older adults living alone reported shorter sleep durations, a larger proportion of poor self-rated health, and poorer ADL than the two other groups. In our study, sleep duration, self-rated health, and ADL were the determinants of depressive symptoms among the three groups; this is consistent with evidence existed [46–48].

Previous studies reported that empty-nest older adults (living alone and living as a couple) had a higher prevalence of depressive symptoms than older adults living with children [22, 23]. However, we found that older adults living as a couple had a lower prevalence of depressive symptoms than those living with children. Along with the progress in society and improvement in their living standards, older adults usually prefer to enjoy their lives [24]. Generally, unlike those living with children [49], older adults living as a couple need not bear the burden of caring for their children; in the meantime, they need not experience higher levels of loneliness compared to those living alone. In addition, spouse support is crucial for older adults’ mental health [50, 51]. In concordance with these prior findings, older adults living as a couple in our sample showed the lowest prevalence of depressive symptoms among the three groups.

According to previous studies [48, 52, 53], the evidence on the relationship between age and depressive symptoms among older adults remains mixed. We found that being ≥ 80 years old was the sole protective factor for depressive symptoms among older adults living with children. Several studies from Asia revealed that fewer older adults were reliant on their children for their daily living, and the net flow of inter-generational support is usually downward, namely from old to the young [49, 54]. Therefore, as older adults’ physical strength gradually decreases with age, the stress of supporting their children or grandchildren could also decrease, potentially having a protective effect on their mental health.

Our findings showed that women were more likely to have depressive symptoms than men among older adults living as a couple and with children, but not among those living alone. In traditional Chinese culture, women usually play a caregiver role and undertake more personal care tasks than men [55]. This care burden may increase depressive symptoms among women [56]. By contrast, as female older adults living alone need not to care for others, they may not experience care-related stress.

Our results also showed that, among older adults living alone, those living in rural areas were more likely to have depressive symptoms than their urban counterparts. Previous studies also showed a higher prevalence of depressive symptoms among rural than urban older adults owing to the gap in economic development, health service resources, and social welfare between rural and urban areas [57, 58]. In addition, family support, especially spousal support, is crucial to older adults’ mental health [50]. Thus, low levels of both social welfare and family support may be associated with vulnerability to depression among rural older adults living alone.

Our data showed that education levels were negatively associated with the occurrence of depressive symptoms among older adults living as a couple and with children. This is consistent with the results of previous studies [9, 59]. However, we found that only the middle school education level was a protective factor for depressive symptoms among those living alone, while the highest educational backgrounds (high school or above) were not. A possible explanation for this phenomenon is the high proportion of women with a lower socioeconomic condition among the older adults living alone in our sample. Cermakova [60] found that women or individuals with a poor socioeconomic condition may not gain a large mental health benefit from education. Several studies revealed that older adults living alone have poorer socioeconomic conditions than those living with others [61, 62]. In this study, 57.9% of older adults were women and 83.5% lived in rural areas. Therefore, the relationship between education levels and depressive symptoms among older adults living alone needs to be further investigated in future study.

Previous studies showed an association between smoking and depressive symptoms, but their findings are mixed [63, 64]. In our study, no smoking was negatively associated with depressive symptoms only among older adults living with children. In this group, as older adults usually live with their children as well as their grandchildren, they may have been greatly concerned about the harmful effects of smoking on the health of the younger generations [65]. This could have, in turn, led them to choose to quit smoking [66], potentially leading to a high risk of developing depressive symptoms [67]. In addition, current smokers reported lower levels of family harmony than non-smokers in prior research [68], and social isolation from family members was associated with more depressive symptoms [69].

Previous findings have shown that those who suffer from chronic diseases have a high risk of depressive symptoms [70, 71]. However, in this study, chronic diseases were associated with the occurrence of depressive symptoms only among older adults living as a couple and with children. As older adults with chronic diseases often need more care, chronic diseases impose a burden on older adults, their families, and society [72, 73]. Then, when older adults living with family members contract chronic diseases, they usually receive more care from their family members than those living alone [24]. However, from the viewpoint of older adults, they often feel fear of being a burden to their families [74]. The guilt of perceiving oneself as a burden could lead older adults to have more depressive symptoms [75]. As older adults who live alone often take care of themselves or request the care of professional caregivers, they may show less guilt in terms of increasing family members’ burden. This may be the mechanism behind our results for this population; however, this finding remains to be further assessed in future research.

We also found that participation in economic activities was a risk factor of depressive symptoms among older adults living alone. Compared with the groups of older adults living as a couple and with children, those living alone may tend to have less financial support, which results in them experiencing more stress from economic pressures and participating in economic activities passively, which in turn impacts their mental health negatively [76].

This study has several limitations. First, as it is a cross-sectional study, the causal relationship between depressive symptoms and its determinants could not be determined. Second, as a survey based on self-reporting, there is a risk of recall bias and inaccurate answers from participants. Third, the CES-D 10 was used to assess depressive symptoms; however, it serves only as a screening tool and cannot be used to diagnose depression [48]. Fourth, this study did not include older adults changed starting their current living arrangement less than 11 months prior to the survey. As such, the short-term effects of living arrangement on depressive symptoms need to be considered in future study [16]. Fifth, due to different reasons for living alone (such as never married or bereavement) may affect older adults’ depressive symptom status, which also need to be considered in future research. Finally, nearly 80% of the sample in this study were rural Chinese older adults, which may limit the generalizability of the findings to all older adults.

## Conclusions

Among the participants, those living as a couple had the lowest prevalence of depressive symptoms, while those living alone had the highest prevalence. The determinants of depressive symptoms differed by living arrangement. Shorter sleep duration, poorer ADL, and poorer self-rated health were the main risk factors for depressive symptoms among participants. Women, those with lower education levels, and those with chronic diseases had a higher risk of depressive symptoms among older adults living as a couple and with children. Smoking was a risk factor of depressive symptoms among older adults living with children; participation in economic activities was a risk factor of depressive symptoms among older adults living alone. We suggest that community health workers, especially those who work in rural areas, should pay increased attention to mental health of older adults living alone. Implementing targeted interventions should be considered not only their living arrangements but also the factors affecting their depressive symptoms.

### Electronic supplementary material

Below is the link to the electronic supplementary material.

Supplementary Material 1

## Data Availability

The datasets analysed for the current study are available in the CHARLS (https://charls.charlsdata.com/pages/data/111/zh-cn.html).

## Abbreviations

ADL: activities of daily living
BMI: body mass index
CES-D 10: 10-item Center for Epidemiologic Studies Depression Scale
CHARLS: China Health and Retirement Longitudinal Study
CI: Confidence intervals
OR: Odds ratios
