# Supplementary material for: Prevalence and determinants of depressive symptoms among community-dwelling older adults in China based on differences in living arrangements: a cross-sectional study
Source: BMC Geriatr. 2023 Oct 10;23:640. doi: 10.1186/s12877-023-04339-6 (PMC10563220; doi:10.1186/s12877-023-04339-6)
Supplement: Supplementary file 1 — Supplementary Material 1 [file 12877_2023_4339_MOESM1_ESM.docx]

STable 1 Multicollinearity Test

| Groups | Variables | VIF |
| --- | --- | --- |
| Older adults living alone | Age | 1.282 |
|  | Sex | 1.585 |
|  | Marital status | 1.115 |
|  | Education level | 1.249 |
|  | Region of living | 1.266 |
|  | Smoking | 1.323 |
|  | Alcohol consumption | 1.193 |
|  | Sleep duration | 1.064 |
|  | Chronic disease | 1.070 |
|  | ADL | 1.204 |
|  | BMI | 1.070 |
|  | Self-rated health | 1.193 |
|  | Economic activity | 1.314 |
| Older adults living as a couple | Age | 1.108 |
|  | Sex | 1.479 |
|  | Marital status | 1.016 |
|  | Education level | 1.257 |
|  | Region of living | 1.269 |
|  | Smoking | 1.262 |
|  | Alcohol consumption | 1.225 |
|  | Sleep duration | 1.047 |
|  | Chronic disease | 1.134 |
|  | ADL | 1.246 |
|  | BMI | 1.077 |
|  | Self-rated health | 1.212 |
|  | Economic activity | 1.276 |
| Older adults living with children | Age | 1.274 |
|  | Sex | 1.766 |
|  | Marital status | 1.322 |
|  | Education level | 1.324 |
|  | Region of living | 1.309 |
|  | Smoking | 1.318 |
|  | Alcohol consumption | 1.236 |
|  | Sleep duration | 1.051 |
|  | Chronic disease | 1.114 |
|  | ADL | 1.208 |
|  | BMI | 1.085 |
|  | Self-rated health | 1.212 |
|  | Economic activity | 1.360 |

Note: BMI, body mass index; ADL, activities of daily living; VIF, variance inflation factors.

STable 2 Hosmer and Lemeshow Test

| Groups | Chi-square | df | *P* |
| --- | --- | --- | --- |
| Older adults living alone | 2.989 | 8 | 0.935 |
| Older adults living as a couple | 11.598 | 8 | 0.170 |
| Older adults living with children | 2.517 | 8 | 0.961 |

STable 3 Association between living arrangement and depressive symptoms among Chinese community-dwelling older adults

| Living arrangement | B | SE | *P* | OR | *95%*CI |
| --- | --- | --- | --- | --- | --- |
| Living alone (ref.) |  |  |  |  |  |
| Living as a couple | -0.514 | 0.149 | **0.001** | 0.598 | (0.447,0.800) |
| Living with children | -0.288 | 0.125 | **0.022** | 0.750 | (0.587,0.958) |

Note: Model adjusted for age, sex, marital status, educational level, region of living, smoking, alcohol consumption, sleep duration, chronic disease, ADL, BMI, self-rated health, and economic activity.
